# Supplementary material for: Immune Responses to SARS-CoV-2 Infection and Vaccine in a Big Italian COVID-19 Hospital: An 18-Month Follow-Up
Source: Vaccines (Basel). 2022 Dec 20;11(1):8. doi: 10.3390/vaccines11010008 (PMC9864433; doi:10.3390/vaccines11010008)
Supplement: Supplementary file 1 [file vaccines-11-00008-s001.zip › vaccines-2081467-supplementary.pdf]

Supplementary material

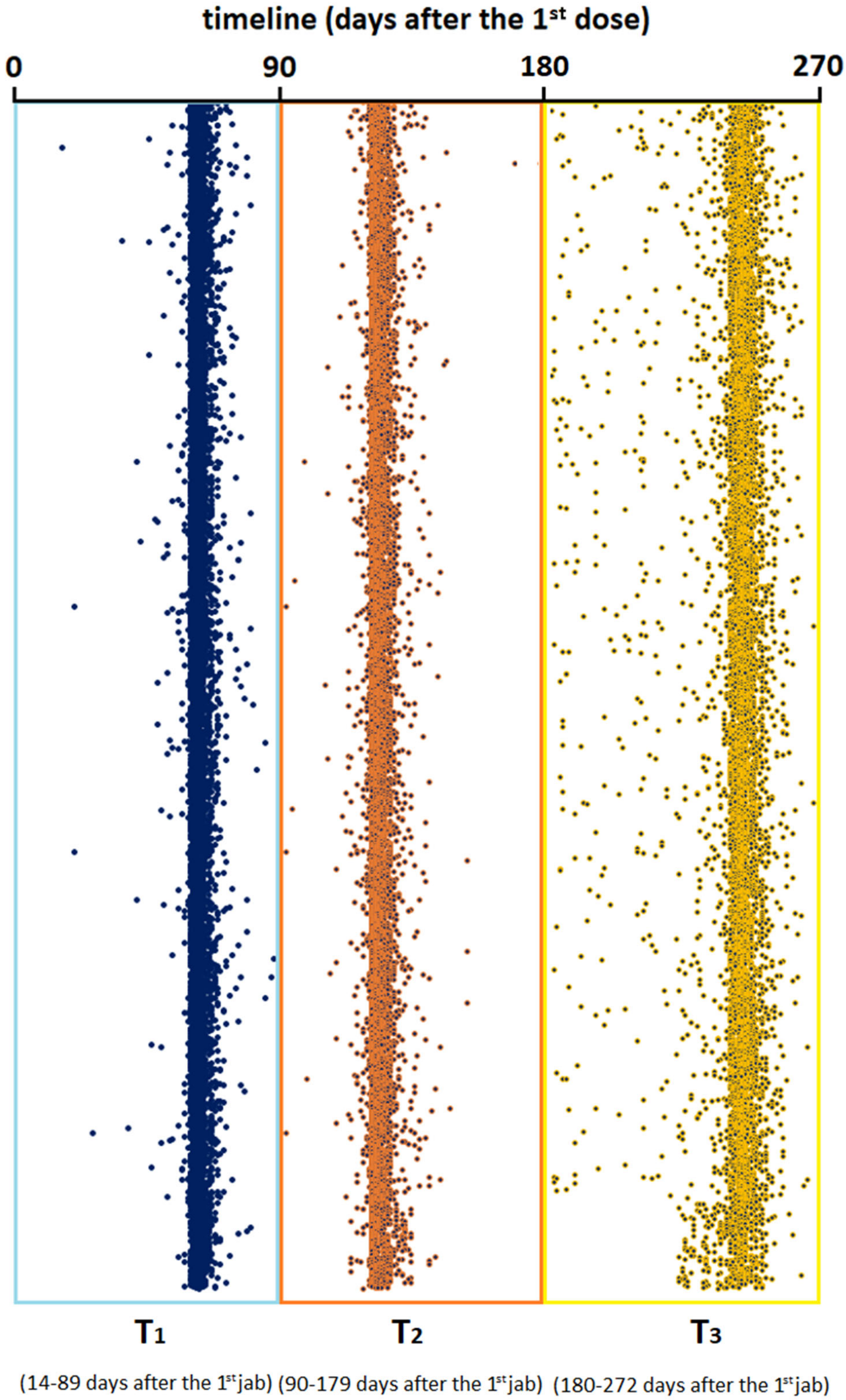

Figure S1. Serum sample collected after vaccination at each stage (T_1 - blue, T_2 - orange, T_3 - yellow). Each dot represents a serum sample collection. Serum samples collected from the same worker at different stages are represented at the same height.

Table S1. Sample description and serological test results. All categorical variables are reported in the following table along with their numbers and percentages. Subgroup total is reported whenever needed. († row percentages; ‡column percentages; HCW healthcare workers; Anti-S: the assay performed revealed and measured anti-Spike antibodies; Anti-N: the assay performed revealed and measured anti-Nucleocapside antibodies; T_0 group includes results obtained from serum samples collected within 24 hours from the 1st jab; T_1 group includes results obtained from serum samples collected between 14 and 89 days since the 1st vaccine jab; T_2 group includes results obtained from serum samples collected between 90 and 179 days since the 1st vaccine jab; T_3 group includes results obtained from serum samples collected 180-272 days after the 1st vaccine jab; denominators for positivizations were obtained from the amount of vaccinated workers who either tested in negative at the beginning of the considered interval and had a serological test at the end of the considered interval; denominators for negativizations (seroreversions) were obtained from the number of vaccinated workers who either tested in positive at the beginning of the considered interval and had a serological test at the end of the considered interval).

Subgroup	Gender† Male (N. 2598, 27.5%)	Gender† Female (N. 6838, 72.5%)	Total N. (%)‡ (N. 9436)
**Age group**			
20-29 years	445 (29.5%)	1062 (70.5%)	1507 (16.0%)
30-39 years	625 30.3%)	1440 (69.7%)	2065 (21.9%)
40-49 years	513 (24.0%)	1628 (76.0%)	2141 (22.7%)
50-59 years	674 (23.2%)	2237 (76.8%)	2911 (30.8%)
over 60 years	341 (42.0%)	471 (58.0%)	812 (8.6%)
**Job title**			
Administrative	328 (33.3%)	657 (66.7%)	985 (10.4%)
Technician	145 (20.7%)	557 (79.3%)	702 (7.4%)
Other HCW	303 (17.6%)	1416 (82.4%)	1719 (18.2%)
Nurse	510 (17.9%)	2345 (82.1%)	2855 (30.3%)
Physician	1160 (43.9%)	1482 (56.1%)	2642 (28.0%)
External workers	152 (28.5%)	381 (71.5%)	533 (5.6%)
**Vaccinated worker**			
Yes	2404 (27.8%)	6244 (72.2%)	8648 (91.6%)
No	194 (24.6%)	594 (75.4%)	788 (8.4%)
**Vaccine jabs**			
1	108 (21.8%)	387 (78.2%)	495 (5.7%)
2	2296 (28.2%)	5857 (71.8%)	8153 (94.3%)
Total	2404 (27.8%)	6244 (72.2%)	8648 (100.0%)
**Type of vaccine**			
Pfizer®	2381 (27.8%)	6185 (72.2%)	8566 (99.1%)
Moderna®	5 (27.8%)	13 (72.2%)	18 (0.2%)
Vaxviria®	11 (21.6%)	40 (78.4%)	51 (0.5%)
Unknown	7 (53.8%)	6 (46.2%)	13 (0.2%)
Total	2404 (27.8%)	6244 (72.2%)	8648 (100.0%)
**Vaccination month**			
December 2020	32 (52.5%)	29 (47.5%)	61 (0.7%)
January 2021	2105 (28.0%)	5400 (72.0%)	7505 (86.8%)
February 2021	131 (31.5%)	285 (68.5%)	416 (4.8%)
March 2021	58 (18.3%)	259 (81.7%)	317 (3.7%)
April 2021	49 (25.1%)	146 (74.9%)	195 (2.3%)
May 2021	29 (18.8%)	125 (81.2%)	154 (1.8%)
Total	2404 (27.8%)	6244 (72.2%)	8648 (100.0%)

Subgroup	Gender† Male (N. 2598, 27.5%)	Female (N. 6838, 72.5%)	Total N. (%)‡ (N. 9436)
**Any positive serological test till vaccination day (cumulative, only for vaccinated workers)**			
Yes	493 (28.9%)	1215 (71.1%)	1708 (19.8%)
No	1911 (27.5%)	5029 (72.5%)	6940 (80.2%)
**1st Serological screening / Apr-Jul 2020 / Anti-S**			
Positive	297 (29.1%)	725 (70.9%)	1022 (13.5%)
Negative	1745 (26.6%)	4818 (73.4%)	6563 (86.5%)
Total	2042 (26.9%)	5543 (73.1%)	7585 (100.0%)
**2nd Serological screening / Oct-Dec 2020 / Anti-N**			
Positive	364 (28.7%)	903 (71.3%)	1267 (16.9%)
Negative	1621 (26.0%)	4616 (74.0%)	6237 (83.1%)
Total	1985 (26.5%)	5519 (73.5%)	7504 (100.0%)
**3rd Serological screening / Jan-Feb 2021 / Anti-N results included in T_0 group**			
Positive	373 (28.6%)	929 (71.4%)	1302 (19.0%)
Negative	1476 (26.5%)	4084 (73.5%)	5560 (81.0%)
Total	1849 (26.9%)	5013 (73.1%)	6862 (100%)
**4th Serological screening / March-April 2021 / Anti-S results included in T_1 group**			
Positive	1920 (27.3%)	5103 (72.7%)	7023 (99.9%)
Negative	3 (50.0%)	3 (50.0%)	6 (0.1%)
Total	1923 (27.4%)	5106 (72.6%)	7029 (100.0%)
**4th serological screening / March-April 2021 /Anti-N results included in T_1 group**			
Positive	398 (28.5%)	999 (71.5%)	1397 (19.7%)
Negative	1537 (27.0%)	4146 (73.0%)	5683 (80.3%)
Total	1935 (27.3%)	5145 (72.7%)	7080 (100.0%)
**5th serological screening / May-June 2021 / Anti-S results included in T_2 group**			
Positive	1746 (26.3%)	4885 (73.7%)	6631 (99.9%)
Negative	2 (33.3%)	4 (66.7%)	6 (0.1%)
Total	1748 (26.3%)	4889 (73.7%)	6637 (100.0%)
**5th serological screening / May-June 2021 / Anti-N results included in T_2 group**			
Positive	359 (27.4%)	953 (72.6%)	1312 (19.7%)
Negative	1391 (26.1%)	3942 (73.9%)	5333 (80.3%)
Total	1750 (26.3%)	4895(14.3%)	6645 (100.0%)
**6th serological screening / Aug-Oct 2021 / Anti-S results included in T_3 group**			
Positive	1803 (26.4%	5017 (73.6%)	6820 (99.9%)
Negative	5 (55.6%)	4 (44.4%)	9 (0.1%)
Total	1808 (26.5%)	5021 (73.5%)	6829 (100.0%)
**6th serological screening / Aug-Oct 2021 / Anti-N results included in T_3 group**			
Positive	369 (27.0%)	999 (73.0%)	1368 (20.0%
Negative	1439 (26.4%)	4017 (73.6%)	5456 (80.0%)
Total	1808 (26.5)	5016 (73.5%)	6824 (100.0%)
**Seroconversions occurred between T_0 and T_1 (Anti-N)**			
Positivization	14/1440 (18.7%)	61/4005 (82.3%)	75/5445 (1.4%)

Subgroup	Gender† Male (N. 2598, 27.5%)	Gender† Female (N. 6838, 72.5%)	Total N. (%)‡ (N. 9436)
Negativization (seroreversion)	5/363 (23.8%)	16/911 (76.2%)	21/1274 (1.6%)
**Seroconversions occurred between T_1 and T_2 (Anti-N)**			
Positivization	9/1381 (22.5%)	31/3919 (77.5%)	40/5300 (0.8%)
Negativization (serorevesion)	2/349 (11.1%)	16/925 (88.9%)	18/1274 (1.4%)
**Seroconversions occurred between T_0 and T_2 (Anti-N)**			
Positivization	20/1320* (19.2%)	84/3851* (80.8%)	104/5171 (2.0%)
Negativization (seroreversion)	7/326 (20.0%)	28/868** (80.0%)	35/1194 (2.9%)
**Seroconversions occurred between T_0 and T_3 (Anti-N)**			
Positivization	19/1291 (15.3%)	105/3770 (84.7%)	124/5061 (2.5%)
Negativization (seroreversion)	20/332 (29.9%)	47/865 (70.1%)	67/1197 (5.6%)
**Seroconversions occurred between T_1 and T_3 (Anti-N)**			
Positivization	9/1349	58/3835	67/5184 (1.3%)
Negativization (seroreversion)	18/352	41/917	59/1269 (4.6%)
**Seroconversions occurred between T_2 and T_3 (Anti-N)**			
Positivization	5/1292 (15.2%)	28/3739 (84.8%)	33/5031 (0.7%)
Negativization (seroreversion)	16/330 (27.2%)	27/902 (62.8%)	43/1232 (3.5%)

Table S2. Distributions of anti-SARS-CoV-2-N titres (U/mL; medians and 1st and 3rd quartiles) in workers stratified by age groups across time (at T_1, T_2 and T_3). P-value is estimated through the bootstrapped ANOVA for repeated measure. Anti-N: antibodies targeting the nucleocapside protein.

	**T_0 Anti-N titres Median, U/mL (IQR)**	**T_1 Anti-N titres Median, U/mL (IQR)**	**T_2 Anti-N titres Median, U/mL (IQR)**	**T_3 Anti-N titres Median, U/mL (IQR)**	**p-value**
Overall	27 (8– 72)	23 (7–67)	20 (6–59)	14 (5–41)	<0.001
Age groups (years)					0.008
20-29	13.6 (5–38)	12 (5–38)	10 (4–32)	8 (3–21)	
30-39	16.2 (5–55)	15.4 (5–49)	13 (4–40)	11 (4–27)	
40-49	25.3 (8–74)	26.0 (8–69)	22 (6–58)	14 (5–41)	
50-59	45.7 (14–96)	37.1 (11–85)	30 (10–76)	21 (7–54)	
60-79	59 (22-109)	50.8 (17–114)	42 (13–108)	27 (11–69)	

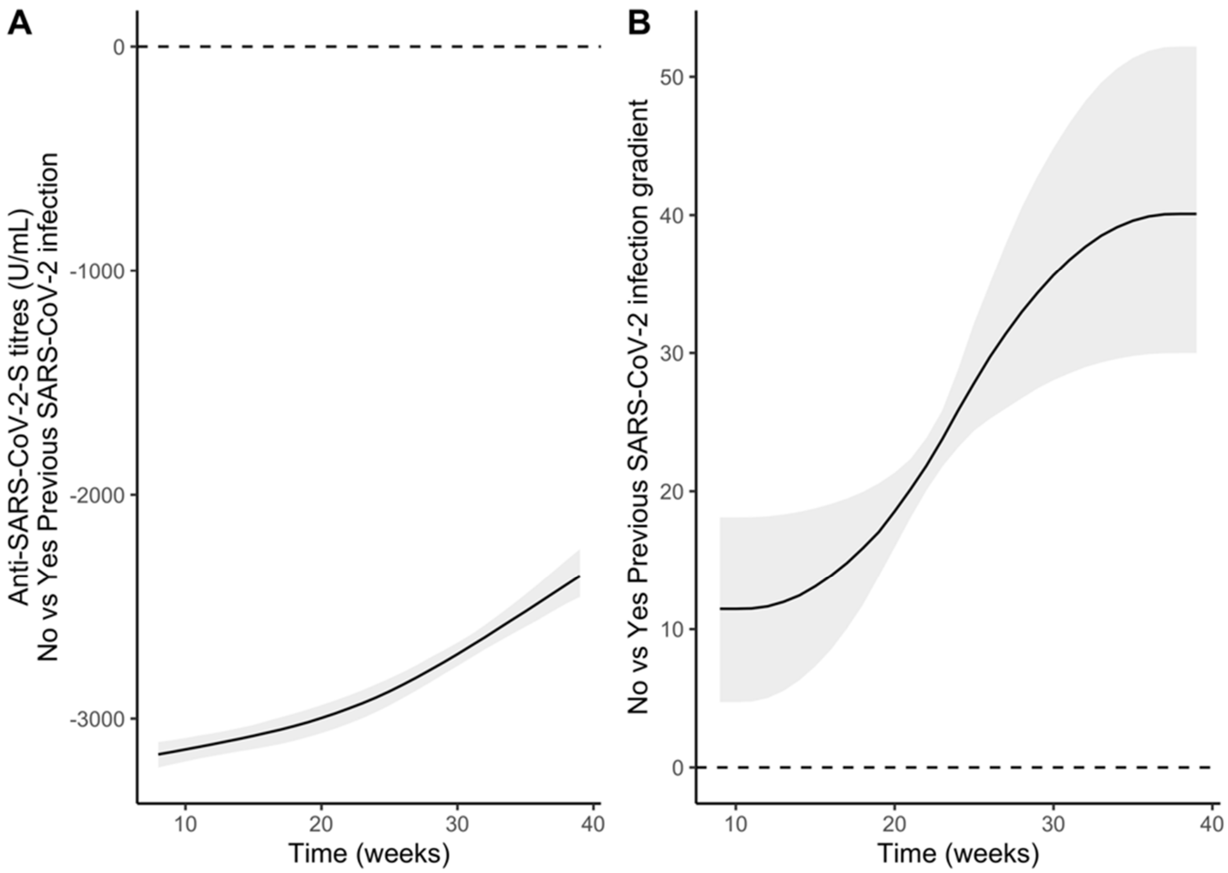

Figure S2. Difference of the anti-S antibody titres (U/mL) (panel A) and difference of the gradient of anti-S antibody titres (panel B) over time between subjects with no pre-vaccine infection and subjects with pre-vaccine infection by SARS-CoV-2. Curves were obtained from the predictions of the bootstrapped linear mixed model adjusted by age and sex.

Table S3. Estimates of the differences and their 95%CI of the anti-S antibody titres (U/mL) and the gradient of anti-S antibody titres between subjects with no pre-vaccine infection and subjects with pre-vaccine infection by SARS-CoV-2 at the average time of the three visits (9, 18 and 35 weeks). Estimates were obtained from the bootstrapped linear mixed model adjusted by age and sex.

**Time**	**Difference estimates (95%CI)**	**Gradient difference estimates (95%CI)**
Week 9	-3148.8 (-3203.9, -3096.6)	11.5 (4.7, 18.1)
Week 18	-3033.2 (-3098.8, -2977.6)	17.1 (13.8, 20.6)
Week 35	-2522 (-2589.7, -2445.7)	39.9 (29.9, 51.9)

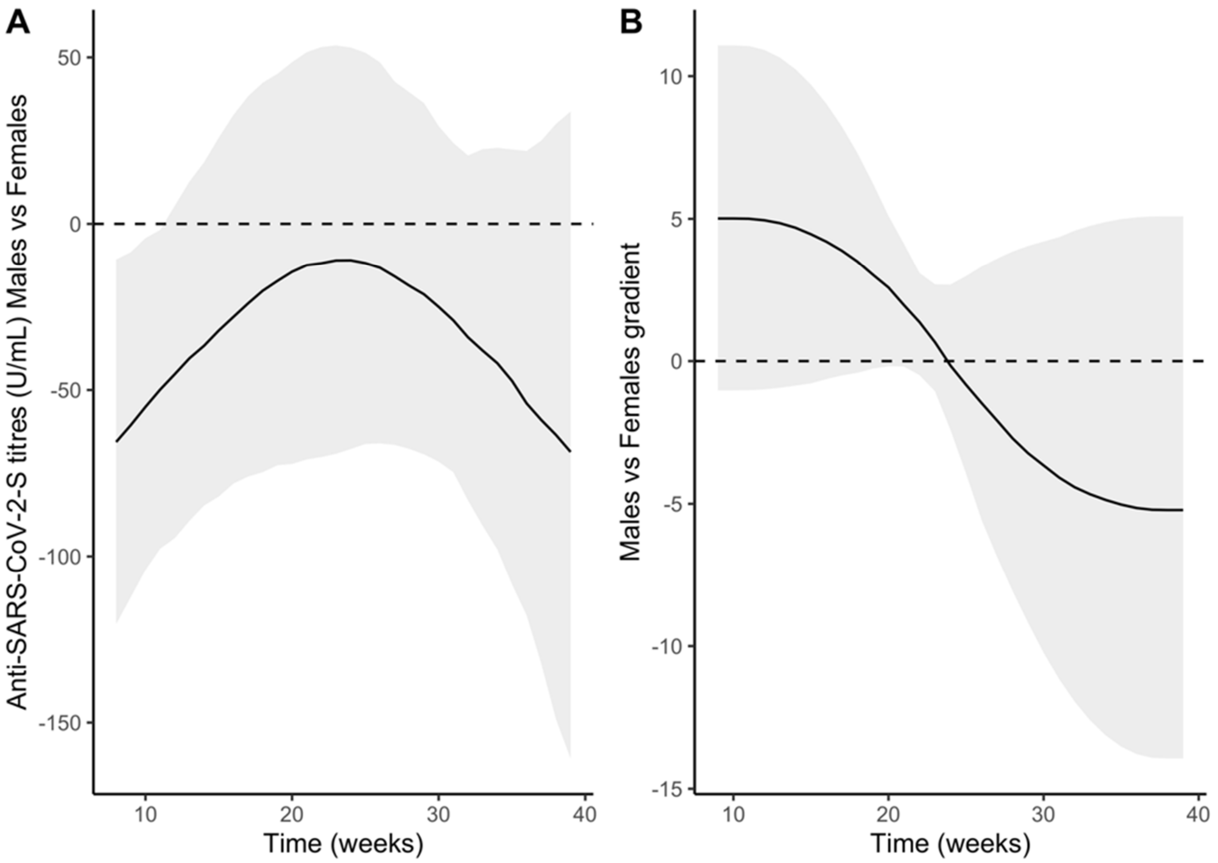

Figure S3. Difference of the anti-S antibody titres (U/mL) (panel A) and difference of the gradient of anti-S antibody titres (panel B) over time between genders. Curves were obtained from the predictions of the bootstrapped linear mixed model adjusted by age and pre-vaccine SARS-CoV-2 infection.

Table S4. Estimates of the differences and their 95%CI of the anti-S antibody titres (U/mL) and the gradient of anti-S antibody titres between genders at the average time of the three visits (9, 18 and 35 weeks). Estimates were obtained from the bootstrapped linear mixed model adjusted by age and pre-vaccine SARS-CoV-2 infection.

Time	Difference estimates (95%CI)	Gradient difference estimates (95%CI)
Week 9	-60.5 (-112.2, -8.5)	5 (-1, 11.1)
Week 18	-20.2 (-74.6, 42.4)	3 (-0.3, 6.2)
Week 35	-47.2 (-108.4, 22.3)	-5.1 (-13.8, 5.1)

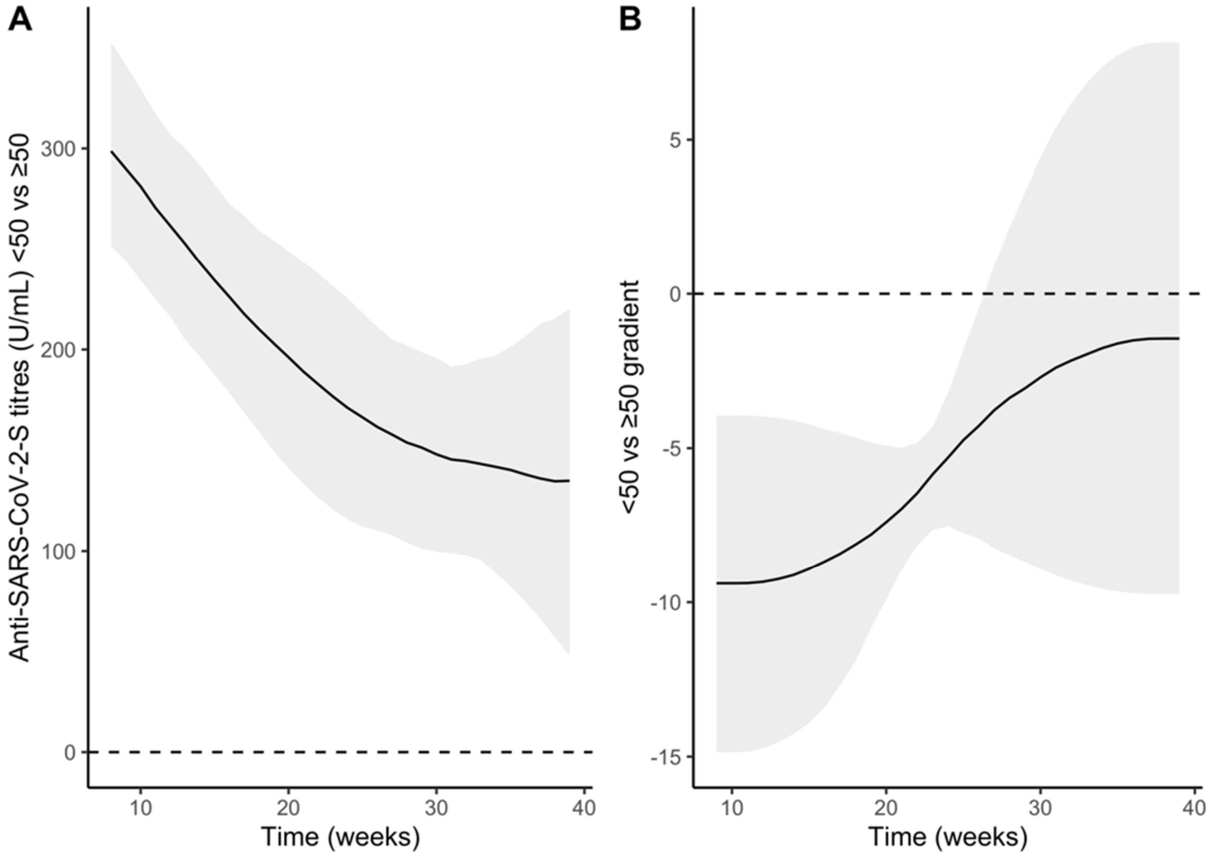

Figure S4. Difference of the anti-S antibody titres (U/mL) (panel A) and difference of the gradient of anti-S antibody titres (panel B) over time between age groups (<50 years old or ≥50 years old). Curves were obtained from the predictions of the bootstrapped linear mixed model adjusted by sex and pre-vaccine SARS-CoV-2 infection.

Table S5. Estimates of the differences and their 95%CI of the anti-S antibody titres (U/mL) and the gradient of anti-S antibody titres between age groups (<50 years old or ≥50 years old) at the average time of the three visits (9, 18 and 35 weeks). Estimates were obtained from the bootstrapped linear mixed model adjusted by sex and pre-vaccine SARS-CoV-2 infection.

Time	Difference estimates (95%CI)	Gradient difference estimates (95%CI)
Week 9	289.9 (244.1, 341.3)	-9.4 (-14.9, -3.9)
Week 18	210.1 (159.2, 259.2)	-7.8 (-10.8, -4.8)
Week 35	140.2 (82.4, 201.3)	-1.5 (-9.7, 8)

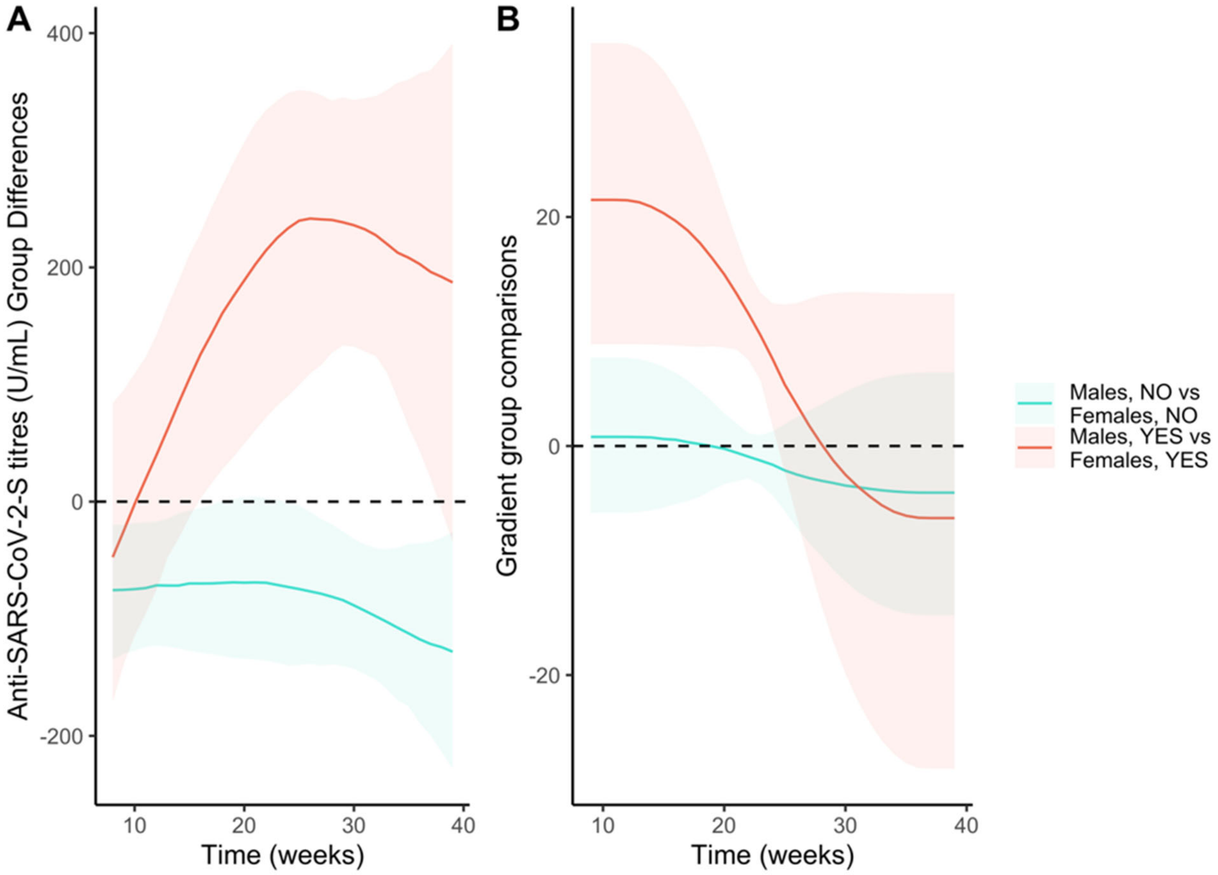

Figure S5. Difference of the anti-S antibody titres (U/mL) (panel A) and difference of the gradient of anti-S antibody titres (panel B) over time between males with no pre-vaccine SARS-CoV-2 infection and females with no pre-vaccine SARS-CoV-2 infection and between males with pre-vaccine SARS-CoV-2 infection and females with pre-vaccine SARS-CoV-2 infection. Curves were obtained from the predictions of the bootstrapped linear mixed model adjusted by age.

Table S6. Estimates of the differences and their 95%CI of the anti-S antibody titres (U/mL) and the gradient of anti-S antibody titres between males with no pre-vaccine SARS-CoV-2 infection and females with no pre-vaccine SARS-CoV-2 infection and between males with pre-vaccine SARS-CoV-2 infection and females with pre-vaccine SARS-CoV-2 infection at the average time of the three visits (9, 18 and 35 weeks). Estimates were obtained from the bootstrapped linear mixed model adjusted by age.

**Comparisons**	**Time**	**Difference estimates (95%CI)**	**Gradient difference estimates (95%CI)**
Males, NO vs Females, NO	Week 9	-75.3 (-129.9, -19.7)	0.8 (-5.8, 7.7)
Males, NO vs Females, NO	Week 18	-69.4 (-131.6, 0.7)	0 (-3.8, 3.9)
Males, NO vs Females, NO	Week 35	-112.6 (-174.8, -36.2)	-4.1 (-14.7, 6.4)
Males, YES vs Females, YES	Week 9	-25.1 (-138.8, 95.1)	21.5 (8.9, 35.2)
Males, YES vs Females, YES	Week 18	160.9 (27.7, 270)	15 (8.6, 21.2)
Males, YES vs Females, YES	Week 35	208.2 (63.8, 360.3)	-6.3 (-28.2, 13.3)

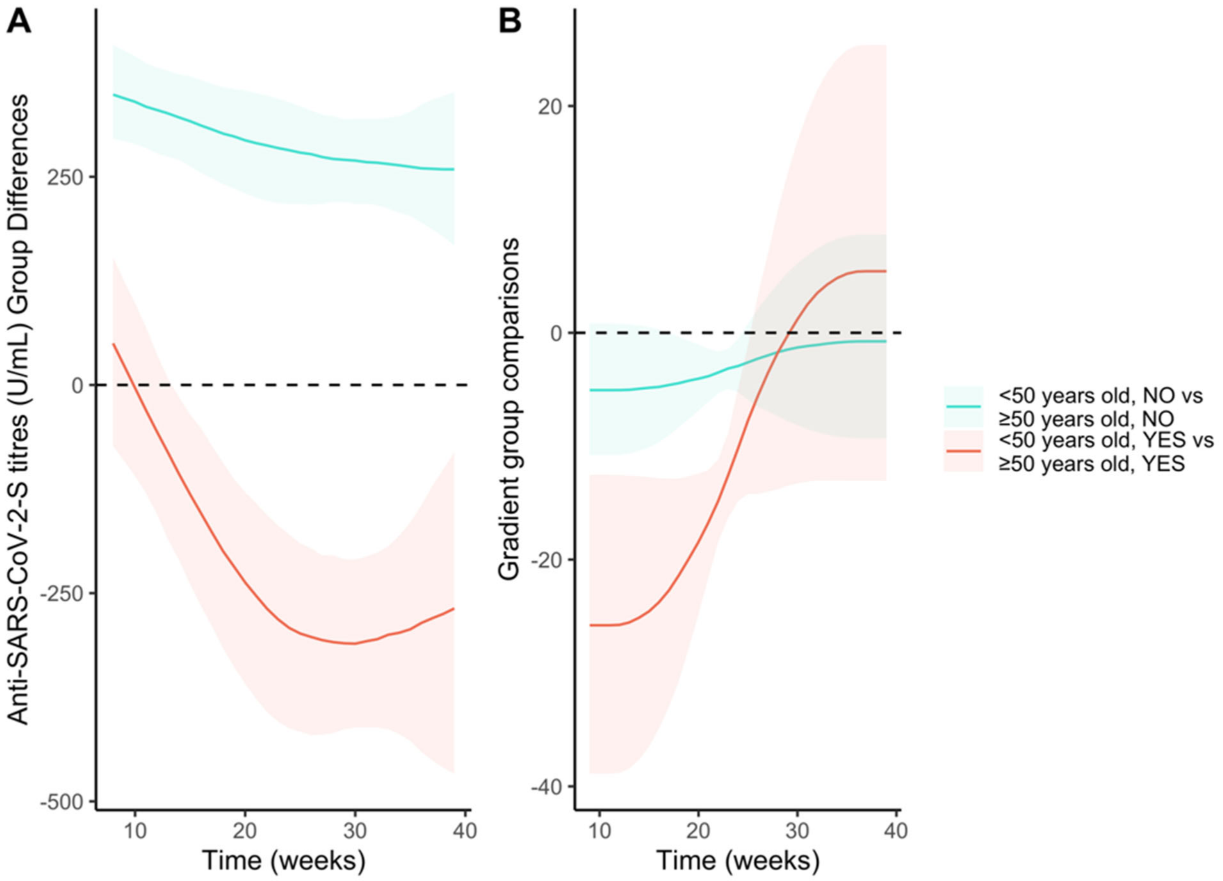

Figure S6. Difference of the anti-S antibody titres (U/mL) (panel A) and difference of the gradient of anti-S antibody titres (panel B) over time between subjects below 50 years old with no pre-vaccine SARS-CoV-2 infection and subjects above 50 years old with no pre-vaccine SARS-CoV-2 infection and between subjects below 50 years old with pre-vaccine SARS-CoV-2 infection and subjects above 50 years old with pre-vaccine SARS-CoV-2 infection. Curves were obtained from the predictions of the bootstrapped linear mixed model adjusted by sex.

Table S7. Estimates of the differences and their 95%CI of the anti-S antibody titres (U/mL) and the gradient of anti-S antibody titres between subjects below 50 years old with no pre-vaccine SARS-CoV-2 infection and subjects above 50 years old with no pre-vaccine SARS-CoV-2 infection and between subjects below 50 years old with pre-vaccine SARS-CoV-2 infection and subjects above 50 years old with pre-vaccine SARS-CoV-2 infection at the average time of the three visits (9, 18 and 35 weeks). Estimates were obtained from the bootstrapped linear mixed model adjusted by sex.

Comparisons	Time	Difference estimates (95%CI)	Gradient difference estimates (95%CI)
<50 years old, NO vs ≥50 years old, NO	Week 9	344.3 (292.9, 401.4)	-5.1 (-10.8, 0.8)
<50 years old, NO vs ≥50 years old, NO	Week 18	301.8 (241.2, 357.3)	-4.2 (-7.6, -0.7)
<50 years old, NO vs ≥50 years old, NO	Week 35	262 (203.3, 327.8)	-0.8 (-9.3, 8.6)
<50 years old, YES vs ≥50 years old, YES	Week 9	23.2 (-90.9, 124.5)	-25.8 (-38.9, -12.5)
<50 years old, YES vs ≥50 years old, YES	Week 18	-199.8 (-316.3, -94)	-18.4 (-24.8, -12.4)
<50 years old, YES vs ≥50 years old, YES	Week 35	-293.4 (-427.2, -165.2)	5.4 (-13, 25.4)

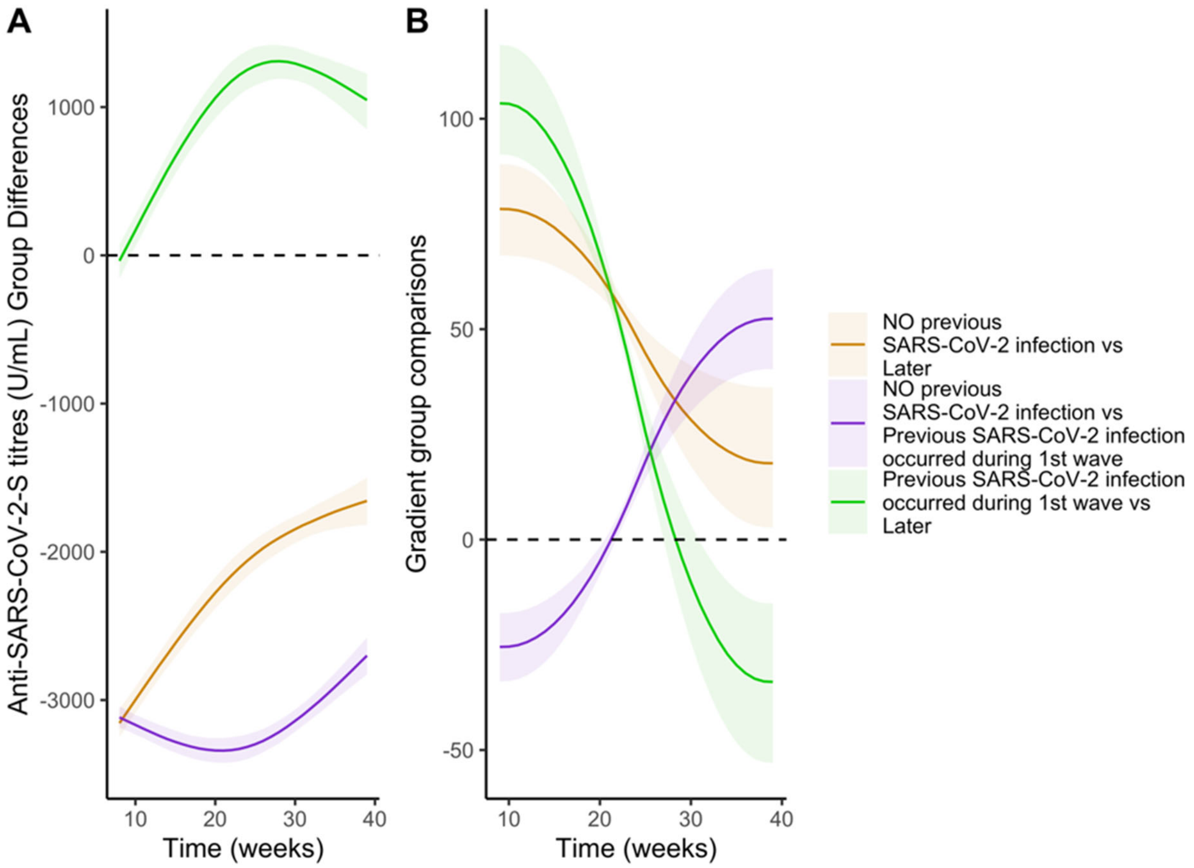

Figure S7. Difference of the anti-S antibody titres (U/mL) (panel A) and difference of the gradient of anti-S antibody titres (panel B) over time between subjects with no pre-vaccine SARS-CoV-2 infection and subjects who experienced a SARS-CoV-2 infection during 1st wave, between subjects with no pre-vaccine SARS-CoV-2 infection and subjects who experienced a SARS-CoV-2 infection later and between subjects who experienced a SARS-CoV-2 infection during 1st wave and subjects who experienced a SARS-CoV-2 infection later. Curves were obtained from the predictions of the bootstrapped linear mixed model adjusted by age and sex.

Table S8. Estimates of the differences and their 95%CI of the anti-S antibody titres (U/mL) and the gradient of anti-S antibody titres between subjects with no pre-vaccine SARS-CoV-2 infection and subjects who experienced a SARS-CoV-2 infection during 1st wave, between subjects with no pre-vaccine SARS-CoV-2 infection and subjects who experienced a SARS-CoV-2 infection later and between subjects who experienced a SARS-CoV-2 infection during 1st wave and subjects who experienced a SARS-CoV-2 infection later at the average time of the three visits (9, 18 and 35 weeks). Estimates were obtained from the bootstrapped linear mixed model adjusted by age and sex.

**Comparisons**	**Time**	**Difference estimates (95%CI)**	**Gradient difference estimates (95%CI)**
NO pre-vaccine SARS-CoV-2 infection vs Pre-vaccine SARS-CoV-2 infection occurred during 1st wave	Week 9	-3142.7 (-3207.4, -3072)	-25.4 (-33.6, -17.4)
NO pre-vaccine SARS-CoV-2 infection vs Pre-vaccine SARS-CoV-2 infection occurred during 1st wave	Week 18	-3327 (-3405.3, -3248.6)	-8.9 (-12.6, -4.5)
NO pre-vaccine SARS-CoV-2 infection vs Pre-vaccine SARS-CoV-2 infection occurred during 1st wave	Week 35	-2910.8 (-2998.4, -2817.9)	51.3 (39.7, 62.8)
NO pre-vaccine SARS-CoV-2 infection vs Later	Week 9	-3077.5 (-3167.6, -2986.9)	78.2 (67.3, 88.7)

NO pre-vaccine SARS-CoV-2 infection vs Later	Week 18	-2406.3 (-2506.1, -2306.9)	62.6 (58, 67)
NO pre-vaccine SARS-CoV-2 infection vs Later	Week 35	-1729.5 (-1845.9, -1618.7)	18.5 (3.4, 36.3)
Pre-vaccine SARS-CoV-2 infection occurred during 1st wave vs Later	Week 9	65.3 (-45.8, 174.5)	101.6 (89.7, 115)
Pre-vaccine SARS-CoV-2 infection occurred during 1st wave vs Later	Week 18	919.1 (790.7, 1040.2)	60.5 (56, 64.9)
Pre-vaccine SARS-CoV-2 infection occurred during 1st wave vs Later	Week 35	1178.6 (1034.4, 1307.3)	-33.7 (-52.9, -15.1)
